# A nomogram to predict the risk of prolonged length of stay following primary total hip arthroplasty with an enhanced recovery after surgery program

**DOI:** 10.1186/s13018-021-02877-6

**Published:** 2021-12-14

**Authors:** Haosheng Wang, Tingting Fan, Wenle Li, Bo Yang, Qiang Lin, Mingyu Yang

**Affiliations:** 1 Department of Orthopedics, Taizhou Central Hospital (Affiliated Hospital To Taizhou College), Donghai Street, Taizhou, 317700 Zhejiang Province People’s Republic of China; 2 Department of Endocrinology, Baoji City Hospital of Traditional Chinese Medicine, Baoji, Shaanxi Province People’s Republic of China; 3 Department of Orthopedics, Xianyang Central Hospital, Xianyang, People’s Republic of China; 4 Clinical Medical Research Center, Xianyang Central Hospital, Xianyang, People’s Republic of China; 5 Department of Orthopedics, Baoji City Hospital of Traditional Chinese Medicine, Baoji, Shaanxi Province People’s Republic of China

**Keywords:** Total hip arthroplasty, Length of stay, Enhanced recovery after surgery, Risk factors, Nomogram

## Abstract

**Background:**

The aim of this study was to identify the risk factors associated with prolonged length of stay (LOS) in patients undergoing primary total hip arthroplasty (THA) managed with an enhanced recovery after surgery (ERAS) program and develop a prediction model for improving the perioperative management of THA.

**Methods:**

In this single-center retrospective study, patients who underwent primary THA in accordance with ERAS from May 2018 to December 2019 were enrolled in this study. The primary outcome was prolonged LOS (> 48 h) beyond the first postoperative day. We collected the clinical patient’s clinical characteristics, surgery-related parameters, and laboratory tests. A logistic regression analysis explored the independent risk factors for prolonged LOS. According to published literature and clinical experience, a series of variables were selected to develop a nomogram prediction model to predict the risk of prolonged LOS following primary THA with an ERAS program. Evaluation indicators of the prediction model, including the concordance index (C-index), the receiver operating characteristic (ROC) curve, calibration curve, and decision curve analysis, were reported to assess the performance of the prediction model. The bootstrap method was conducted to validate the performance of the designed nomogram.

**Results:**

A total of 392 patients were included in the study, of whom 189 (48.21%) had prolonged LOS. The logistics regression analysis demonstrated that age, sex, hip deformities, intraoperative blood loss, operation time, postoperative Day 1 (POD) hemoglobin (Hb), POD albumin (ALB), and POD interleukin-6 (IL-6) were independent risk factors for prolonged LOS. The C-index was 0.863 (95% CI 0.808 to 0.918) and 0.845 in the bootstrapping validation, respectively. According to the results of the calibration, ROC curve, and decision curve analyses, we found that the nomogram showed satisfactory performance for prolonged LOS in this study.

**Conclusions:**

We explored the risk factors for prolonged LOS following primary THA with an ERAS program and developed a prediction model. The designed nomogram was expected to be a precise and personalized tool for predicting the risk and prognosis for prolonged LOS following primary THA with an ERAS program.

## Background

With the world's population aging, the demand for total hip arthroplasty (THA) is rising. Since the 1920s, THA has become one of the most effective treatments for many hip conditions. Enhanced recovery after surgery (ERAS) is a concept that has become increasingly popular for arthroplasty surgery over the last ten years [1–3]. The goals of ERAS are to promote faster recovery, reduce postoperative complications, shorten the length of stay (LOS), reduce the burden of medicine on society, and improve patient quality of life and satisfaction [4]. Henrik Kehlet [5], a Danish surgeon, has extensively addressed this complex pathophysiological phenomenon in the perioperative period since the late 1980s in collaboration with all surgical disciplines. As such, he used existing basic knowledge to change the perspectives in clinical practice. Today, the ERAS program has been considered an efficient and cost-effective use of health resources.

Notably, there is a huge difference between developed and developing countries in ERAS implementation. Therefore, based on the database of the project group for the "National Health and Family Planning Commission's public-benefit project: the safety and effect assessment of joint arthroplasty" and evidence-based medicine, a consensus (hereafter, "consensus") has been reached, to provide a reference for medical teams [6–9]. ERAS for patients undergoing primary THA has been in place in our hospital since May 2018. Painless surgery and fast recovery are common pursuits of surgeons and THA patients. Painless surgery and fast recovery are common pursuits of surgeons and THA patients. The focus of ERAS in TKA is to improve surgical techniques and optimize perioperative management, including the reduction of trauma and hemorrhage, optimization of pain and blood management, prevention of infection and deep vein thrombosis, and promotion of early mobilization. However, some patients are slow to discharge, which leads to delays [10]. This observation led us to investigate the related risk factors for prolonged LOS and reduce the costs, and in-hospital complications. At present, studies have not focused on establishing a prediction model for the risk of prolonged LOS after primary THA with an ERAS program. Therefore, it is necessary to fully understand the clinical characteristics of patients who underwent primary THA with an ERAS program to identify the risk factors for prolonged LOS. A well-developed clinical nomogram can be used to predict individual outcomes, which is beneficial to both patients and clinicians.

Thus, the aim of this study was to develop a predictive model by analyzing the data in our hospital to determine the risk for prolonged LOS following primary THA with an ERAS program.

## Methods

### Inclusion and exclusion criteria

Inclusion criteria: (1) Primary THA; (2) strict implementation of ERAS measures in consensus; (3) clear consciousness and no communication barriers; and (4) volunteered to participate. The exclusion criteria were as follows: (1) severe hematological diseases; (2) serious coexisting diseases such as heart, brain, and lung; and (3) mental illness or communication difficulties. This retrospective study was approved by the ethical and research committee of Taizhou Central Hospital (Project ID: 202009795N). All patients provided written informed consent.

### Patients and data collection

A total of 392 patients who underwent primary THA strictly according to the ERAS consensus at the Department of Orthopedics, Taizhou Central Hospital from May 2018 to Dec 2019 were retrospectively reviewed. This retrospective study was approved by the ethical and research committee of Taizhou Central Hospital (Project ID: 202009795N). The primary THA operations were performed by the same surgeon following standard procedures through a posterolateral approach under general anesthesia. Preoperative pre-emptive analgesia usually chosen from NSAIDs or selective COX-2 inhibitors. Patients with high preoperative anxiety may be given diazepam and Stilnox and, if needed, the anxiolytic drugs olanzapine, escitalopram and sertraline. In addition, 15–20 mg/kg TXA was administered intravenously (IV) prior to skin incision and 1–2 g TXA was applied topically before closing the incision. No drainage tube was placed after the operation. Postoperative pain was treated with multimodal analgesics, including ice, NSAIDs, selective COX‐2 inhibitors, morphine, pethidine and oxycodone. The specific use of analgesics can be adjusted by the attending physician according to the patient's specific condition.

Demographic and clinical parameters, including age, sex, body mass index (BMI), Harris score, proportion of hip deformities, hip joint mobility, and preoperative visual analogue score (VAS), were collected. Preoperative routine laboratory tests, included blood tests, blood electrolytes, liver function, and kidney function. Operation-related parameters including the American Society of Anesthesiologists (ASA) parameters, operation time, anesthesia time, intraoperative infusion volume, and intraoperative blood loss were recorded. A routine hematological examination was performed 1 day after surgery. Thus, we calculated the Hb change rate on Day 1 after surgery. POD Hb change = (preoperative Hb-POD Hb)/preoperative Hb * 100%, where POD represents one day after surgery. In parallel, we recorded the VAS score on the day of surgery and Day 1 after surgery, out-of-bed activity time after surgery, total infusion volume on the day of surgery, and postoperative infusion volume. In this study, patients with LOS ≤ 48 h and > 48 h were the A and B groups, respectively. Moreover, parameters of postoperative complications, including postoperative nausea and vomiting (PONA), urinary tract infection, intermuscular vein thrombosis venous, deep vein thrombosis (DVT), delayed wound healing, superficial infection and deep infection, were collected.

### Discharge criteria and follow-up

Patients who meet the following four criteria are judged to have recovered and can be released and discharged. (I): The patient's vital signs were stable, fever was not observed, spirit and appetite had returned to the preoperative level and stool was normal; (II) the incision was dry with no signs of infection, such as erythema and induration; and (III) hip pain after surgery was not obvious, could be effectively relieved by oral analgesics, did not affect the patient's sleep and functional exercises, and had a resting pain VAS score of < 3 points and a pain VAS score of < 5 points when active. The oral medications often used for postoperative pain are nonsteroidal anti-inflammatory drugs (NSAIDs) or selective COX‐2 inhibitors, such as diclofenac, loxoprofen sodium, celecoxib, and rofecoxib, combined with opioid analgesics for severe pain, including oral tramadol or oxycodone. After meeting the discharge criteria, patients were discharged home. Patient postoperative follow-up was conducted regularly at the outpatient department of Taizhou Central Hospital. Outpatient follow-up at was performed at 1 week, 2 weeks, 1 month, 2 months, 3 months, 6 months and 12 months after the operation. During the follow-up at 2 weeks after operation, the stitches were removed according to the healing of the incision, and venous thrombosis of the lower extremities was examined by color Doppler ultrasound.

### Statistical analysis and model construction

Continuous variables are presented as the means ± standard deviations or as medians and interquartile ranges (depending on the data distribution) and were evaluated using the Student’s t test or Mann–Whitney U test, as appropriate. Categorical variables were grouped and compared using the *χ*^2^ test or Fisher’s exact test. Forward step‐wise multivariable logistic regression analysis, including covariates identified in univariate logistic analysis, was used to identify independent factors associated with prolonged LOS following primary THA with an ERAS program. Meanwhile, the odds ratio (OR) and 95% confidence intervals (CIs) were reported. According to the results of the regression coefficients of independent variables, an individual nomogram prediction model for prolonged LOS was established. The designed nomogram model was internally validated using bootstrap sampling (1000 resamples). The area under the receiver-operating characteristic curve (AUC) or c statistic was used to assess the discrimination of the nomogram in receiver operating characteristic (ROC) curves. An AUC of 0.5 indicated no diagnostic performance; 0.5–0.7 indicated inaccuracy in discrimination; 0.7–0.9 indicated moderate performance; and > 0.9 indicated excellent performance. Calibration was evaluated using a calibration plot to compare the relationship between the observed outcome frequencies versus the predicted outcomes. A decision curve analysis (DCA) was performed to assess the clinical net benefit. Analyses were performed with SPSS software (SPSS standard, version 26.0; SPSS, Inc.) and R version 4.0.1 (R Foundation for Statistical Computing). The nomogram was created in R software using the "rms" package. In this study, *P* < 0.05 was considered statistically significant.

## Results

Details of the patient population are shown in Table 1. A total of 392 patients were included, with 206 (52.6%) males and 186 (47.4%) females, the mean age 61.2 ± 7.0 years. Among the 392 patients, 203 patients had LOS ≤ 48 h, i.e., Group A; 189 patients had LOS > 48 h, i.e., Group B. Among the A and B group, there were statistically significant differences were observed in age (*P* < 0.001), sex (*P* = 0.04), hip deformities (*P* = 0.035), hip joint mobility (*P* = 0.008), preoperative VAS (*P* = 0.012), preoperative Hb (*P* = 0.003), operation time (*P* < 0.001), anesthesia time (*P* < 0.001), intraoperative infusion volume (*P* < 0.001), out of bed activities time (*P* < 0.001), postoperative infusion volume (*P* < 0.001), total infusion volume on the day of surgery (*P* < 0.001), VAS score on the day of surgery (*P* < 0.001), POD VAS score (*P* < 0.001), POD ALB (*P* < 0.001), POD IL-6 (*P* < 0.001), POD total blood loss (*P* = 0.044), and POD Hb change rate (*P* < 0.001). Significant differences were not observed in remaining parameters between the A and B groups. Among the 392 patients in this study, we observed postoperative complications in both the A group (LOS ≤ 48 h) and B group (LOS > 48 h). Significant differences were not observed in PONA (*P* = 0.821), urinary tract infection (*P* = 0.031), intermuscular vein thrombosis venous (*P* = 0.245) or deep infection (*P* = 0.381) between the two groups. However, delayed wound healing, superficial infection, DVT, and total complications were significantly lower in the A group (LOS ≤ 48 h) than B group (LOS ≤ 48 h). Detailed data on the postoperative complications are presented in Table 2.

**Table 1:** Characteristic at baseline between A and B group

	Total	A group	B group	*P* value
Number of patients	392	203	189	
Age (years)	61.2 (7.0)	58.0 (6.8)	64.6 (5.5)	< 0.001
Sex (%)				
Female	186 (47.4)	75 (36.9)	111 (58.7)	0.04
Male	206 (52.6)	128 (63.1)	78 (41.3)	
BMI (kg/m^2^)	25.2 (1.8)	25.0 (1.6)	25.3 (2.0)	0.373
Harris score	44.2 (15.2)	45.4 (12.8)	42.8 (17.4)	0.523
Hip deformities (%)				
No	334 (85.2)	157 (77.3)	177 (93.7)	0.035
Yes	58 (14.8)	46 (22.7)	12 (6.3)	
Hip joint mobility (°)	223.6 (28.0)	230.0 (27.1)	216.8 (27.3)	0.008
Preoperative VAS	6.0 (2.0)	5.6 (1.9)	6.5 (2.1)	0.012
Preoperative Hb (g/L)	134.7 (16.8)	139.0 (12.9)	130.0 (19.2)	0.003
Preoperative ALB (g/L)	44.6 (5.1)	44.0 (5.2)	45.3 (4.9)	0.227
ASA (%)				
I	18 (4.6)	15 (7.4)	3 (1.6)	0.337
II	213 (54.3)	119 (58.6)	94 (49.7)	
III	150 (38.3)	66 (32.5)	84 (44.4)	
IV	11 (2.8)	3 (1.5)	8 (4.2)	
Operation time (min)	74.4 (16.4)	67.8 (12.9)	81.6 (16.7)	< 0.001
Anesthesia time (min)	95.1 (22.3)	87.6 (18.5)	103.2 (23.2)	< 0.001
Intraoperative infusion volume (ml)	372.0 [334.0, 438.0]	337.0 [317.0, 354.0]	440.0 [414.0, 475.0]	< 0.001
Intraoperative blood loss (ml)	182.0 [146.0, 210.0]	182.0 [151.0, 203.0]	183.0 [145.0, 222.0]	0.442
Out of bed activities time (h)	16.4 [13.1, 20.9]	13.6 [11.2, 15.8]	20.9 [17.5, 23.7]	< 0.001
Postoperative infusion volume (ml)	502.0 [474.0, 537.0]	485.0 [466.0, 512.0]	529.0 [481.0, 585.0]	< 0.001
Total infusion volume on the day of surgery (ml)	1050.1 (127.4)	962.4 (80.1)	1144.2 (98.3)	< 0.001
VAS score on the day of surgery	2.8 (0.9)	2.5 (0.9)	3.2 (0.8)	< 0.001
Day 1 after surgery VAS score	3.3 (1.0)	2.7 (0.5)	3.9 (1.1)	< 0.001
POD Hb (g/L)	121.6 (13.7)	123.1 (14.9)	120.1 (12.2)	0.237
POD ALB (g/L)	39.1 (4.6)	40.7 (4.6)	37.4 (4.1)	< 0.001
POD CRP (mg/L)	18.2 (5.2)	18.3 (5.4)	18.1 (5.1)	0.936
POD IL-6 (pg/ml)	20.8 (8.1)	16.5 (6.4)	25.5 (7.2)	< 0.001
POD total blood loss 1 day after surgery (ml)	308.8 (58.7)	298.5 (55.7)	319.8 (59.9)	0.044
POD Hb change rate (%)	9.0 [8.0, 11.0]	8.0 [6.0, 9.0]	10.0 [9.0, 12.0]	< 0.001

A group: LOS ≤ 48 h group; B group: LOS > 48 h group; BMI, body mass index; VAS: visual analogue score; Hb: hemoglobin; ALB: albumin; ASA: American Society of Anesthesiologists; CRP: C-reactive protein; IL-6: interleukin-6; POD: one day after surgery

POD Hb change = (Preoperative Hb − POD Hb)/Preoperative Hb * 100%

**Table 2:** Comparison of variables between the A group and B group

Parameters	A group (n = 203)	B group (n = 189)	*P* value
*Complications (%)*			
PONA	11 (5.42%)	13 (6.88%)	0.821
Urinary tract infection	1 (0.49%)	4 (2.12%)	0.031
Intermuscular vein thrombosis	3 (1.48%)	3 (1.59%)	0.245
DVT	0 (0.00%)	4 (2.12%)	0.0165
Delayed wound healing	0 (0.00%)	5 (2.65%)	0.0071
Superficial infection	0 (0.00%)	6 (3.17%)	< 0.001
Deep infection	0 (0.00%)	1 (0.53%)	0.381
Total	15 (7.39%)	36 (19.05%)	< 0.001

A group: LOS ≤ 48 h group; B group: LOS > 48 h group; PONA: postoperative nausea and vomiting; DVT: deep vein thrombosis

**Fig. 1:**
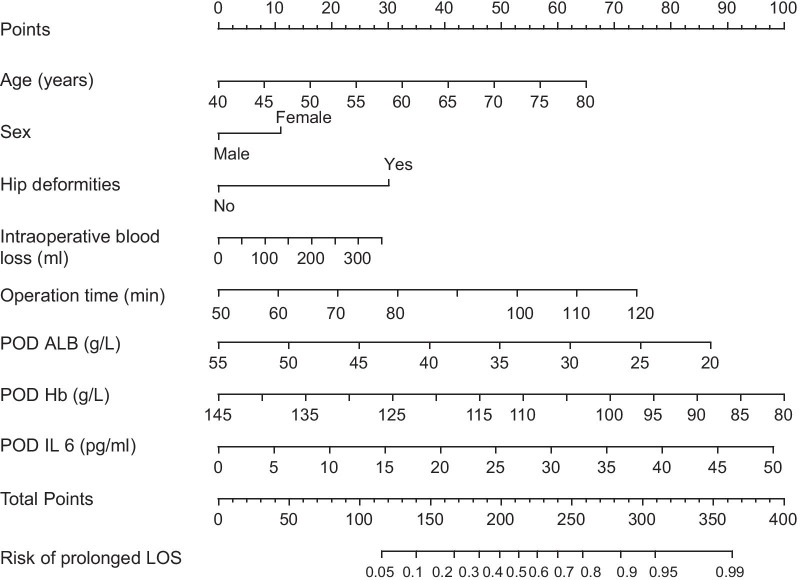
Nomogram to predict the risk of prolonged LOS following primary THA with an ERAS program. LOS: length of stay; THA: total hip arthroplasty; ERAS: enhanced recovery after surgery

In the multivariate logistic regression, the following eight factors were independent risk factors for prolonged LOS (Table 3) (*P* < 0.05): age (odds ratio [OR] = 2.315, 95% confidence interval [CI] = 1.490–3.140, *P* = 0.042), sex (OR 0.305, 95% CI 0.256–0.354, *P* = 0.039), hip deformities (OR 0.296, 95% CI 0.098–0.894, *P* = 0.031), operation time (OR 1.135, 95% CI 1.068–1.202, *P* = 0.008), intraoperative blood loss (OR 1.798, 95% CI 1.645–1.951, *P* = 0.028), POD Hb (OR 0.955, 95% CI 0.924–0.987, *P* = 0.006), POD ALB (OR 0.689, 95% CI 0.656–0.722, *P* = 0.026), and POD IL-6 (OR 1.312, 95% CI 1.063–1.620, *P* = 0.011). Afterward, a nomogram was developed according to the logistic regression analysis (Fig. 1).

**Table 3:** Univariate and multivariate logistic regression model analyses of prolonged LOS following primary THA with an ERAS program

	Univariate analysis		Multivariate analysis	
	OR (95% CI)	P value	OR (95% CI)	P value
Age (years)	1.598 (1.177–2.019)	0.019	2.315 (1.490–3.140)	0.042
Sex (%)	0.765 (0.589–0.941)	0.163	0.305 (0.256–0.354)	0.039
BMI (kg/m2)	1.152 (0.973–1.363)	0.105	NA	
Harris score	0.995 (0.978–1.013)	0.594	NA	
Hip deformities (%)	0.297 (0.139–0.637)	0.002	0.296 (0.098–0.894)	0.031
Hip joint mobility (°)	1.625 (1.013–2.237)	< 0.001	NA	
Preoperative VAS	1.214 (1.035–1.425)	0.017	NA	
Preoperative Hb (g/L)	0.974 (0.956–0.991)	0.003	NA	
Preoperative ALB (g/L)	1.018 (0.963–1.075)	0.53	NA	
ASA (%)	1.432 (0.877–2.338)	0.151	NA	
Operation time (min)	1.014 (0.997–1.030)	0.101	NA	
Anesthesia time (min)	1.018 (1.001–1.034)	0.032	NA	
Intraoperative infusion volume (ml)	2.032 (1.709–2.355)	< 0.001	1.798 (1.645–1.951)	0.028
Intraoperative blood loss (ml)	1.305 (1.185–1.425)	< 0.001	1.135 (1.068–1.202)	0.008
Out of bed activities time (h)	1.151 (1.074–1.233)	0.028	NA	
Postoperative infusion volume (ml)	1.007 (1.001–1.012)	0.013	NA	
Total infusion volume on the day of surgery (ml)	1.004 (1.002–1.007)	0.001	NA	
VAS score on the day of surgery	1.540 (1.062–2.232)	0.023	NA	
Day 1 after surgery VAS score	1.512 (1.138–2.009)	0.004	NA	
POD Hb (g/L)	0.951 (0.928–0.975)	< 0.001	0.955 (0.924- 0.987)	0.006
POD ALB (g/L)	0.548 (0.481–0.615)	< 0.001	0.689 (0.656–0.722)	0.026
POD CRP (mg/L)	0.971 (0.917–1.029)	0.316	NA	
POD IL 6 (pg/ml)	1.485 (1.268–1.739)	< 0.001	1.312 (1.063- 1.620)	0.011
POD total blood loss 1 day after surgery (ml)	1.002 (0.997–1.007)	0.424	NA	
POD Hb change rate (%)	1.077 (1.038–1.118)	< 0.001	NA	

LOS > 48 h group; THA: total hip arthroplasty; ERAS: enhanced recovery after surgery; BMI, body mass index; VAS: visual analogue score; Hb: hemoglobin; ALB: albumin; ASA: American Society of Anesthesiologists; CRP: C-reactive protein; IL-6: interleukin-6; POD: one day after surgery. OR: odds ratio; CI, confidence interval; NA, not available

POD Hb change = (Preoperative Hb − POD Hb)/Preoperative Hb * 100%

**Fig. 2:**
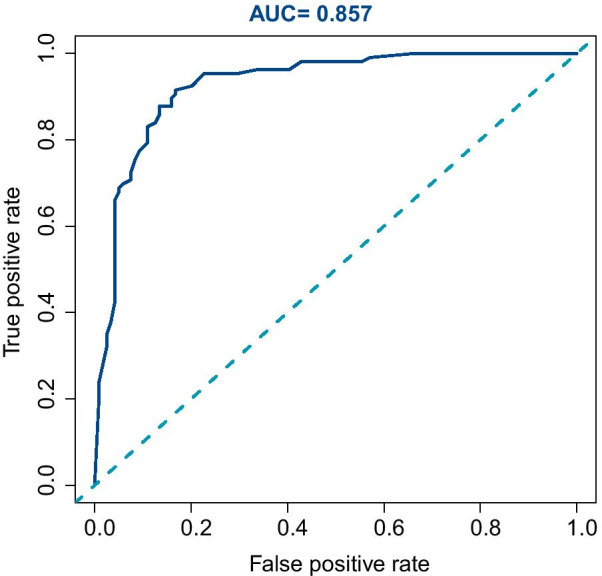
Receiver operating characteristic curve analysis—model validation

**Fig. 3:**
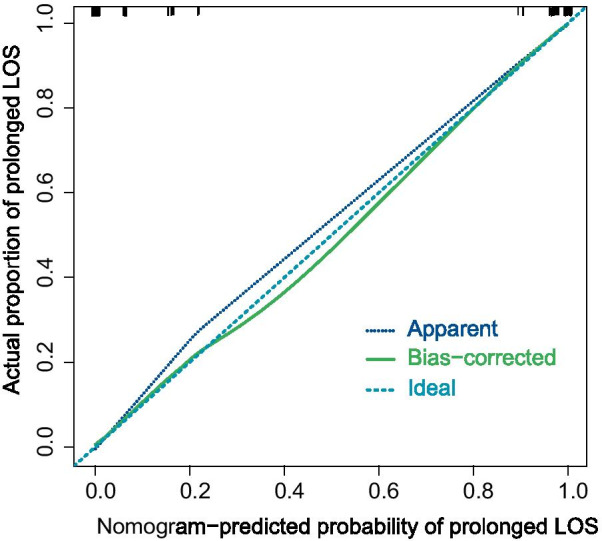
Calibration plot for the nomogram. The apparent and bias corrected values are close to each other, which means that the nomogram has good predictive performance

**Fig. 4:**
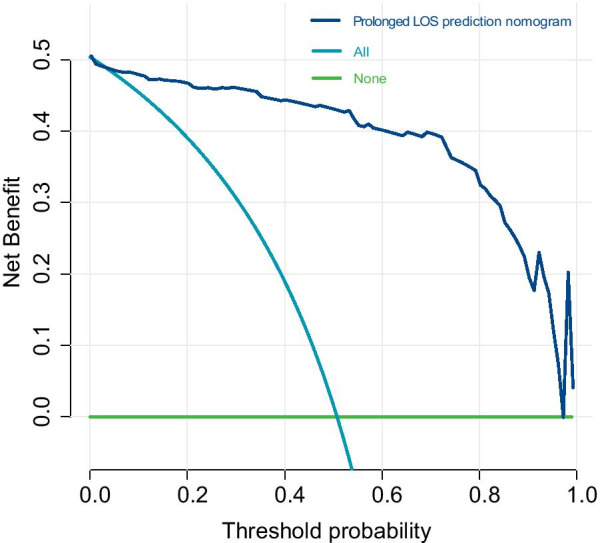
The decision curve analysis (DCA) for the constructed radiomics nomogram model. The X and Y axes represent the threshold probability and net benefit, respectively. Dark blue indicates the hypothesis that all patients had prolonged LOS. The blue line represents the hypothesis that no patients had prolonged LOS

To evaluate the performance of the prediction model, several indicators were reported in this work. The area under the curve (AUC) of the receiver operating characteristic (ROC) curve (Fig. 2) of the model was 0.857 (95% CI, 0.815 to 0.898), indicating that the discrimination performance of the model was satisfactory. Additionally, the calibration curve demonstrated good agreement between the observed probability of prolonged LOS in this study (Fig. 3). Meanwhile, the C-index of the nomogram was 0.863 (95% CI 0.808 to 0.918), while that by bootstrapping validation was 0.845 (bootstrap = 1000). To identify the potential clinical benefit of the designed nomogram, a decision analysis (DCA) was performed in this dataset. The DCA is demonstrated in Fig. 4 and suggested that the clinical net benefit of this risk prediction nomogram in a range of risk thresholds (0.03 to 1.00) was higher than that of all-screening or no-screening strategies.

## Discussion

LOS is an essential indicator for assessing the effectiveness of ERAS implementation in developed countries, such as in Europe and America [4, 11]. The ERAS program was initially introduced in Denmark, although its use has slowly become more widespread in Europe and America. The total LOS of THA and TKA patients has been reduced from 4–12 d in the past to 2–4 d now, which significantly accelerates the postoperative recovery of patients, saves medical resources, reduces the risk of surgery and improves patient safety and satisfaction [12, 13]. Due to a lack of a comprehensive hierarchical diagnosis and treatment system and relatively adequate medical resources, preoperative examination and preparation are refined by family doctors, community hospitals, or outpatient clinics, and most patients are admitted on the day of surgery and transferred soon after surgery to a rehabilitation facility with the necessary accelerated rehabilitation measures [14, 15]. Thus, the LOS may not reflect the total LOS but the postoperative LOS.

Compared with developed countries, patients in China tend to visit top-level hospitals without having adequate preoperative workup due to the uneven distribution of medical resources [16]. The process of standardized diagnosis and treatment has been challenging due to the shortage of medical resources, which has resulted in longer preoperative preparation times. Of potentially greater concern, perhaps, is that most patients have difficulty obtaining adequate referrals for rehabilitation after discharge and are instead discharged home directly [17]. Despite the implementation of ERAS protocols in many medical centers, their effect on reducing LOS remains limited. However, little is known about the possible risk factors for prolonged LOS following primary THA with an ERAS program, and a relatively reliable tool for predicting prognosis is lacking. Thus, we screened and identified risk factors for prolonged LOS and developed a nomogram based on a retrospective study. Bootstrapping was the preferred approach for internal validation. In this work, we identified risk factors associated with prolonged LOS as follows: age, sex, hip deformities, intraoperative blood loss, operation time, POD Hb, POD ALB, and POD IL-6.

A total of 392 patients who achieved consensus were included in the present study, with a mean postoperative LOS of 3.4 ± 1.5 d. Moreover, there was no mortality, and no serious postoperative complications occurred. The patients recovered well after the operation. During the follow-up 3 months after the operation, the excellent and good Harris score rate was 100% (392/392). In this study, 51.79% (203/392) of patients were discharged within 48 h after surgery. The point of 48 h postoperative was set because most of the literature reported LOS ≤ 2 d as an important assessment index of THA in the ERAS program, and the patients in their study were admitted on the day of surgery.

Previously, several studies reported that age is a significant predictor for THA/TKA LOS. In a study reported by Sibia et al. [18], the proportion of patients undergoing THA with a strictly implemented ERAS protocol with a prolonged LOS of more than 1 d was up to 1.8 times greater in patients aged 70 years than in those aged 60 years. We cautiously speculate that this may be related to the fact that elderly patients have more comorbidities, are in a relatively poorer general condition, and require more care. ElBitar et al. [19] reviewed the data of patients undergoing THA under ERAS implementation and found that patients over 65 years of age and especially over 80 years of age had a prolonged LOS compared to patients under 65 years of age. We believe the possible reason for this is the slower functional recovery after major surgery in the elderly, which prolongs LOS. Despite this lack of evidence, the study identified sex as a risk factor for prolonged LOS in this study. Previous studies have reported that there was an association between female patients and prolonged LOS in THA/TKA [20, 21]. Katz et al. [22] a questionnaire survey found that female patients had a relatively more advanced disease state and poorer lower limb function at the time of surgery, which may have contributed to the longer recovery time required for female patients.

Interestingly, in this report, preoperative combined hip deformities were identified as a risk factor for prolonged LOS, which was similar to the conclusion of the study by Zhang et al. [23]. We cautiously speculate that this may be because this type of patient had a long relative history, and the visits often occurred at an advanced stage of the disease. In addition, a proportion of patients had poor muscle strength in the lower limbs, which caused recovery to be relatively difficult and lead to a longer surgery time, thus leading to a slow recovery after surgery and prolonged LOS. The ASA classification and Charlson Comorbidity Index (CCI) are widely accepted parameters for the assessment of comorbidities. Many previous studies have shown that a higher number of comorbidities in patients undergoing THA/TKA corresponds to a higher the risk of prolonged LOS [14, 24, 25]. However, it is worth noting that several issues must be treated with caution such as how to include the types of comorbidities and how to measure the number of comorbidities. Such issues are controversial and will require further study in the future. Therefore, it is important to remain cautious.

In this investigation, intraoperative indicators such as intraoperative blood loss and operation time were identified as risk factors for LOS > 48 h after THA with an ERAS program. In general, intraoperative transfusion volume and intraoperative blood loss were positively correlated with operation time. More importantly, most of these conditions are more challenging for postoperative recovery because of they are associated with more complex surgery, more bleeding, and increased intraoperative transfusion volume. Previous studies have shown that as the duration of surgery increases, the risk of prolonged LOS also increases [26]. Therefore, according to the designed nomogram, we believe that skilled surgical techniques occupy an important position in reducing LOS and optimizing the surgical operation technique to minimize the operation time is beneficial to the patient's postoperative recovery and reduces LOS. Similarly, we observed that POD Hb was identified as a risk factor for prolonged LOS. This point is easy to understand because POD Hb often accurately reflects blood loss during the operation. A lower POD Hb indicates a higher perioperative blood loss. Therefore, prompt postoperative review of blood work and intervention at the appropriate time is warranted.

Certain postoperative laboratory parameters, including POD ALB and POD IL 6, were identified as risk factors for prolonged LOS. Patients with low postoperative ALB levels are at higher risk of wound exudation, hematoma, and poor wound healing, resulting in late bedtime activity and poor exercise outcomes. Several studies have confirmed that the late start of functional exercise of the lower extremity after surgery and poor functional exercise of the lower extremity in the early postoperative period are risk factors for prolonged LOS in patients with THA/TKA [26, 27]. This may be the reason for the prolonged LOS due to low ALB levels on postoperative Day 1. Another unexpected finding of our study was that POD IL 6 was identified as a risk factor for prolonged LOS. IL 6 is a major inflammatory cytokine regulated by HuR binding to mRNA. Interleukin (IL)‐6 is a major inflammatory cytokine regulated by HuR binding to mRNA. In previous studies IL 6 was considered to be an important proinflammatory factor in cells [27]. Although IL 6 extensively involved in many pathophysiological processes, few papers have reported its effect on LOS after THA. We speculate that surgery and anesthesia may cause elevated IL 6; however, clarifying the molecular biological mechanism will require a large number of future studies to verify this conclusion.

Data on complications indicated that postoperative complications had important effects on prolonged LOS, which suggests that complication prevention, early diagnosis, and appropriate treatment measures have an important impact on clinical outcomes. Although complications were not the most important predictors included in the nomogram model, complications are certainly an important factor worthy of separate study in the future.

Here, we designed a model for predicting the risk of prolonged LOS following primary THA with an ERAS program. The nomogram can demonstrate the key parameters graphically and individually to access the incidence of risk of prolonged LOS. Such work will enable accurate patient evaluation and management when encountered in clinical practice. More importantly, the nomogram may provide opportunities for improving perioperative management and the ERAS program in primary THA.

Various limitations were observed in this investigation. First, the investigation included a retrospective data analysis, which might include unknown confounders, meaning that selection and detection bias cannot be completely avoided. Second, there was a lack of external validation for our proposed model, especially in other regions and countries. Third, the ERAS program followed in this study has only been applied in some parts of China; thus, more collaborative studies in more centers are needed to validate it in the future. Finally, we should focus on the impact of complications on prolonged LOS.

## Conclusions

Overall, this study developed a nomogram tool for predicting prolonged LOS following primary THA with an ERAS program. However, the designed cannot effectively discriminate patients with different prolonged LOS risks. The tool has great potential to aid surgeons in stratifying patient risk and provides a reference for improving ERAS programs. The performance of the nomogram was validated using bootstrap method. The developed nomogram is purely academic thus far; however, we plan to integrate it into the information system of our hospital for prospective validation.

## Data Availability

The dataset supporting the conclusions of this article is available on request from the corresponding author.

## Abbreviations

THA: Total hip arthroplasty
ERAS: Enhanced recovery after surgery
BMI: Body mass index
VAS: Visual analogue score
Hb: Hemoglobin
ALB: Albumin
ASA: American Society of Anesthesiologists
CRP: C-reactive protein
IL-6: Interleukin-6
POD: One day after surgery
AUC: Area under the receiver operating characteristic curve
POD: One day after surgery
PONA: Postoperative nausea and vomiting
DVT: Deep vein thrombosis
OR: Odds ratio
CI: Confidence interval
NA: Not available
